# Mitral valve repair with artificial chords: Tips and tricks

**DOI:** 10.1111/jocs.17076

**Published:** 2022-11-02

**Authors:** Michele Di Mauro, Giorgia Bonalumi, Ilaria Giambuzzi, Pietro Messi, Marco Cargoni, Domenico Paparella, Roberto Lorusso, Antonio M. Calafiore

**Affiliations:** ^1^ Department of Cardio‐Thoracic Surgery, Heart & Vascular Centre, Maastricht University Medical Centre (MUMC) Cardiovascular Research Institute Maastricht (CARIM) Maastricht The Netherlands; ^2^ Department of Cardiac Surgery IRCCS Monzino Cardiology Center Milan Italy; ^3^ DISCCO (Dipartimento di Scienze Cliniche e di Comunità) University of Milan Milan Italy; ^4^ Department of Cardiac Surgery Istituto Clinico Sant'Ambrogio Milan Italy; ^5^ Department of Cardiac Anesthesia Mazzini Hospital Teramo Italy; ^6^ Department of Medical and Surgical Sciences, Division of Cardiac Surgery University of Foggia Foggia Italy; ^7^ Division of Cardiac Surgery, Santa Maria Hospital GVM Care & Research Bari Italy; ^8^ Department of Cardiovascular Diseases Gemelli Molise Campobasso Italy

**Keywords:** cardiovascular pathology, clinical review, valve repair/replacement

## Abstract

Mitral valve regurgitation (MR) is a common valvular disorder occurring in up to 10% of the general population. Mitral valve reconstructive strategies may address any of the components, annulus, leaflets, and chords, involved in the valvular competence. The classical repair technique involves the resection of the prolapsing tissue. Chordal replacement was introduced already in the '60, but in the mid '80, some surgeons started to use expanded polytetrafluoroethylene (ePTFE) Gore‐Tex sutures. In the last years, artificial chords have been used also using transcatheter approach such as NeoChord DS 1000 (Neochord) and Harpoon TSD‐5. The first step is to achieve a good exposure of the papillary muscles that before approaching the implant of the artificial chords. Then, the chords are attached to the papillary muscle, with or without the use of supportive pledgets. The techniques to correctly implant artificial chords are many and might vary considerably from one center to another, but they can be summarized into three big families of suturing techniques: single, running or loop. Regardless of how to anchor to the mitral leaflet, the real challenge that many surgeons have taken on, giving rise to some very creative solutions, has been to establish an adequate length of the chords. It can be established based on anatomically healthy chords, but it is important to bear in mind that surgeons work on the mitral valve when the heart is arrested in diastole, so this length could fail to replicate the required length in the full, beating heart. Hence, some surgeons suggested techniques to overcome this problem. Herein, we aimed to describe the current use of artificial chords in real‐world surgery, summarizing all the tips and tricks.

## 1 INTRODUCTION

Mitral regurgitation (MR) is a common valvular disease that occurs in up to 10% of the general population.^1^ It is also the second most frequent indication for heart valve surgery in Europe.^2^

There are two pathways of MR, primary and secondary, and the indications for treatment vary accordingly.

In case of primitive MR, mitral valve (MV) should be, whenever possible, repaired,^2^ as it is associated to better outcomes than surgical replacement.^3^

Mitral valve reconstructive strategies can involve any of the MV components such as the annulus, the leaflets, and chordae.

The classical repair technique encompasses the resection of the prolapsing tissue, the “French Correction”.^4^ Chordal replacement was introduced as early as 1960, when surgeons used silk and nylon.^5^, ^6^ Frater and colleagues^7^ used glutaraldehyde fixed bovine pericardium to replace chordae tendineae in a small number of patients.

Finally, in the mid‐1980s, some surgeons began using expanded polytetrafluoroethylene (ePTFE) Gore‐Tex sutures^8^, ^9^

In the last years, the concept of “respect rather than resect” has taken hold, so the implantation of artificial chords to anchor the leaflets to the papillary muscles has been more widely used.^10^, ^11^ Alongside this concept, the publication of satisfactory long‐term results, with 20‐year freedom from reoperation ranging from 74% to 92%,^12^, ^13^ contributed to the popularity of this surgical approach. Moreover, in the last years, artificial chords have been used also using transcatheter approaches such as NeoChord DS 1000 (Neochord) and Harpoon TSD‐5 (Edwards Lifescience), and ChordArt (CoreMedic).^14^, ^15^

Herein, we aimed to describe the current use of artificial chords in real‐world surgery, summarizing all the tips and tricks.

## 2 ARTIFICIAL CHORDS, STEP‐BY‐STEP

### 2.1 Exposure of the papillary muscles

The first step is to obtain a good exposure of the papillary muscles, which is of paramount importance before implanting the artificial cords. Erlebach et al.^16^ proposed an elegant way to push aside both MV leaflets. The authors used a standard valve prosthesis calibrator (more often a 29‐mm one) to get a clear view of the papillary muscles. Other surgeons^17^ introduced a nickel‐titanium leaflet retractor that can be easily rolled into a cylindrical shape with a diameter of 15 mm and grasped with a long needle holder. Once introduced into the MV annulus, releasing the needle holder opens the retractor spontaneously so that the MV leaflets are pushed to the sides.

### 2.2 The attachment of the cords to the papillary muscles

In most cases, surgeons prefer to use a pledget or small pericardial patch as a support to avoid any injury to the tip of the papillary muscle (PM),^18^ otherwise a figure‐of‐eight suture or simply a U‐stich is used.^19^

### 2.3 The attachment of the cords to the mitral valve leaflets

Techniques for implanting artificial cords are many and can vary greatly from center to center. However, they can be summarized into three major families of suturing techniques: single strings,^20^, ^21^ running,^9^ or loop strings.^22^

A) **Single strings**: In the first technique, the ePTFE suture is passed through the free margin of the flap and anchored to the fibrous part of the PM. At the free margin of the leaflet, the suture is passed with both ends, which are then secured with knots. At midterm follow‐up, freedom from reintervention was 96 ± 4%, with only one patient undergoing reintervention due to hemolysis, but at the time of reintervention the artificial cords were found to be intact. In the same study, freedom from recurrent mitral regurgitation was 94 ± 4%.^23^
B) **Running technique**: The suture is passed through the fibrous portion of the PM and the ends are tied together, leaving one arm end longer than the other. The longer arm is passed once or twice through the leaflet, 4‐5 mm apart at the free margin. Then, the same arm is brought inside the ventricle and passed back into the head of the PM. In this way, numerous pairs of new chords are obtained (also known as the multiple loop technique).^24^ Recently, the results at 20‐year follow‐up have been reported, showing a cumulative reintervention and MR recurrence rate of 4.2% and 14.1%, respectively.
C) **Loop technique and its modifiications**: Multiple loops of predetermined length are anchored to a pledget placed on the PM, and each loop is tied to the free margin of the prolapsed segment with knotting on the ventricular side to avoid distortion of the free margin of the leaflet.^22^ The Leipzig group^25^ recently published the results of their technique, with a 10‐year freedom from reoperation of 97 ± 1%. A number of modifications to the standard loop technique have been proposed over the years^26^, ^27^: the use of small anchors to facilitate eventual length adjustment after hydrostatic testing,^26^ or the use of small pieces of paper as wide as the desired length of the loop, which are tightened around it.^27^

### 2.4 The chordal length

The great challenge for surgeons is to establish an appropriate chordal length.

Ibrahim et al.^28^ tried to classify the different methods into a few groups: fixed length with or without caliper, anatomic length, and adjustable length.

**Figure 1:**
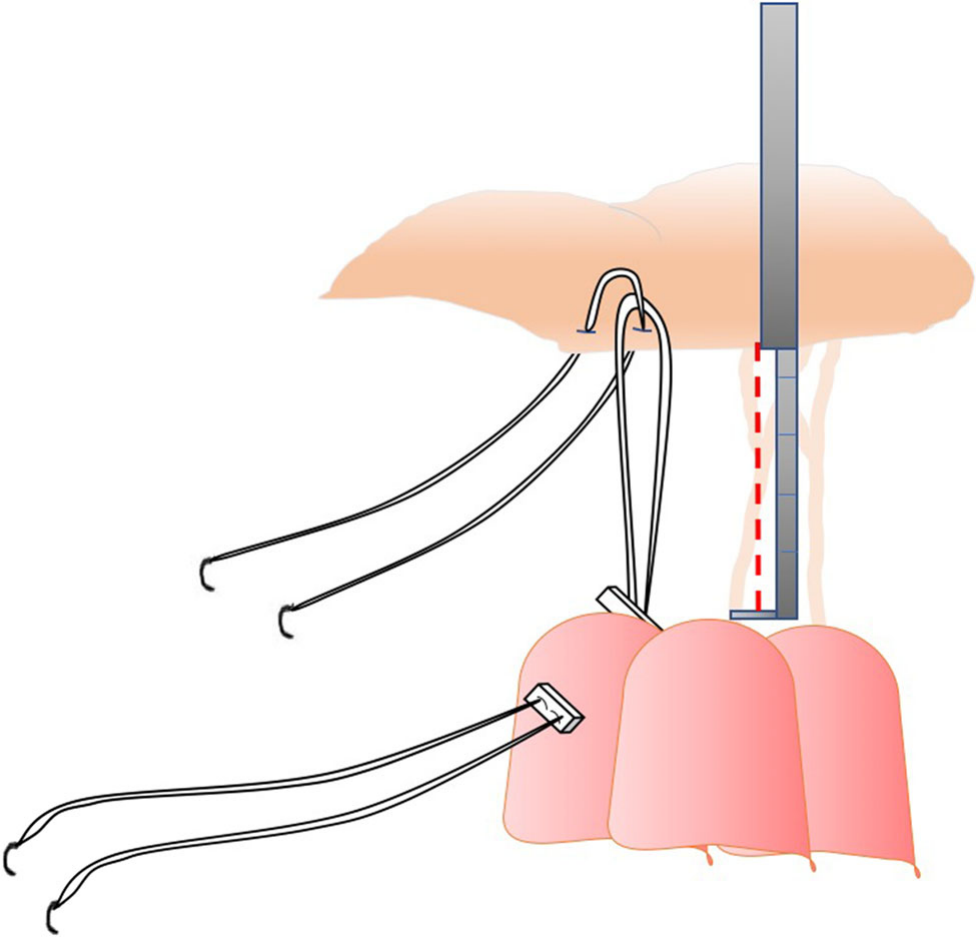
The loop technique proposed by von Oppell and Mohr^22^ (see text).

**Figure 2:**
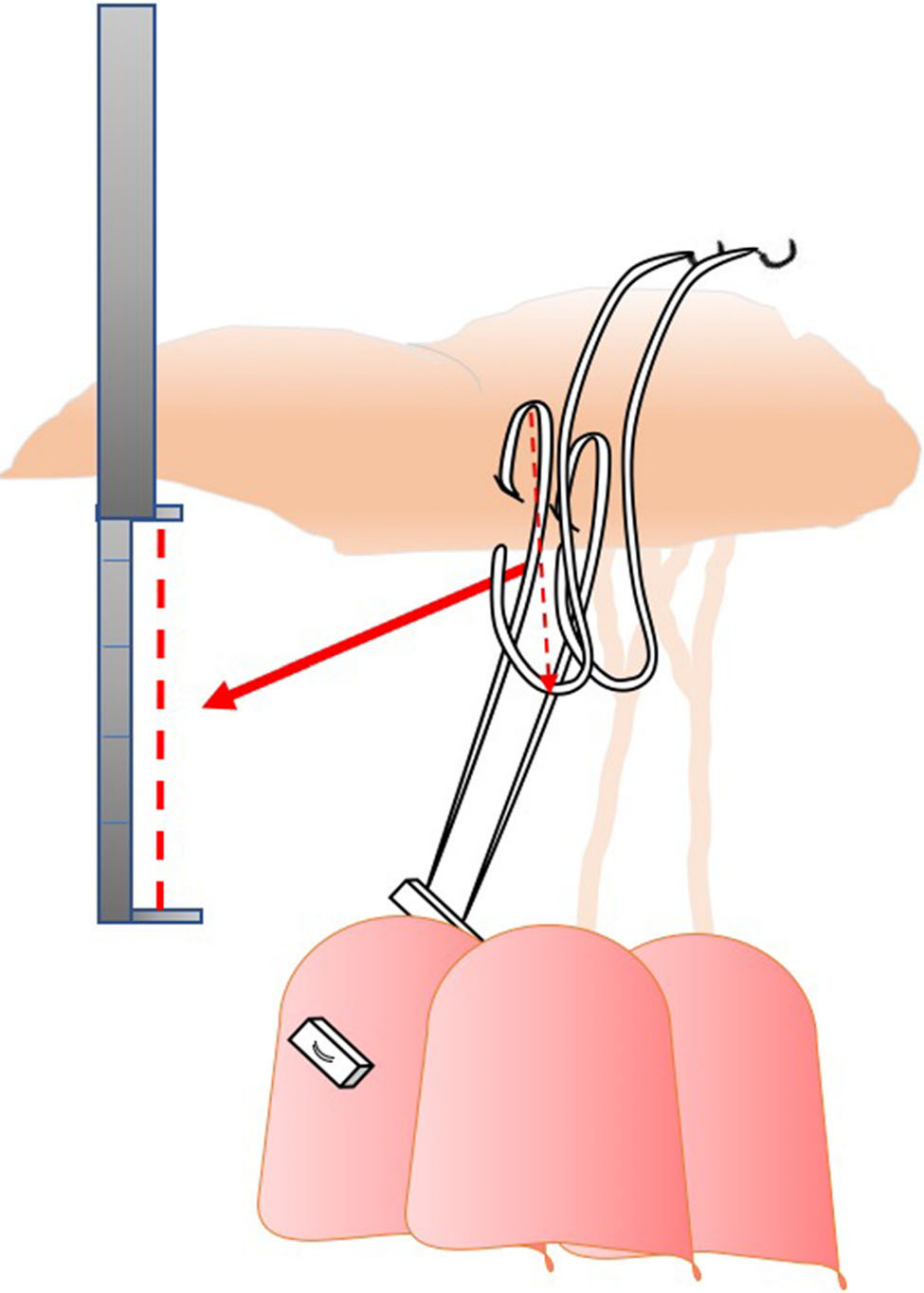
A different use of a caliper was proposed by Doi et al.^27^ (see text).

**Figure 3:**
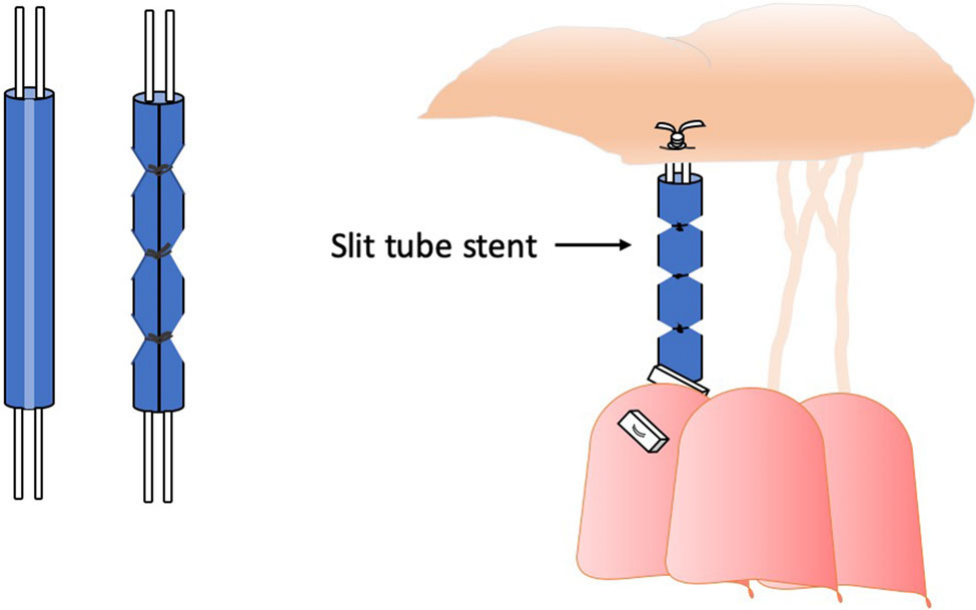
Loops are tied at a predetermined length temporarily fixing them at a specific length using a slit tube, as proposed by Chang et al.^32^

**Figure 4:**
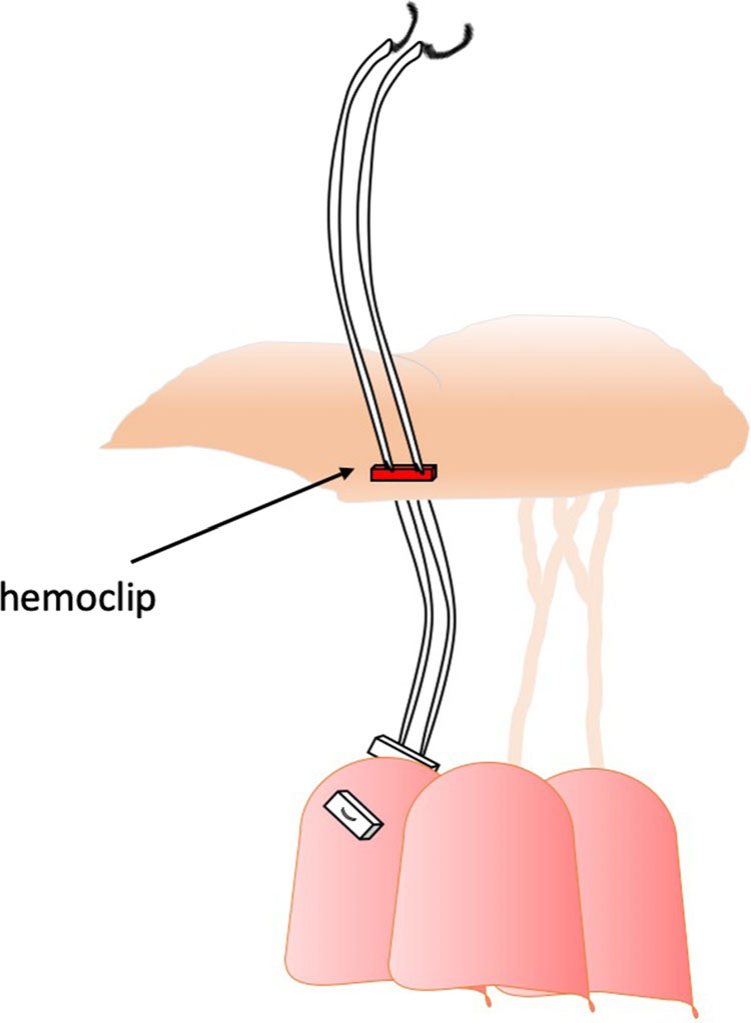
Chan et al.^35^ proposed the use of a covered clip holding the chords at the correct length, allowing them to be tied without movement.

**Figure 5:**
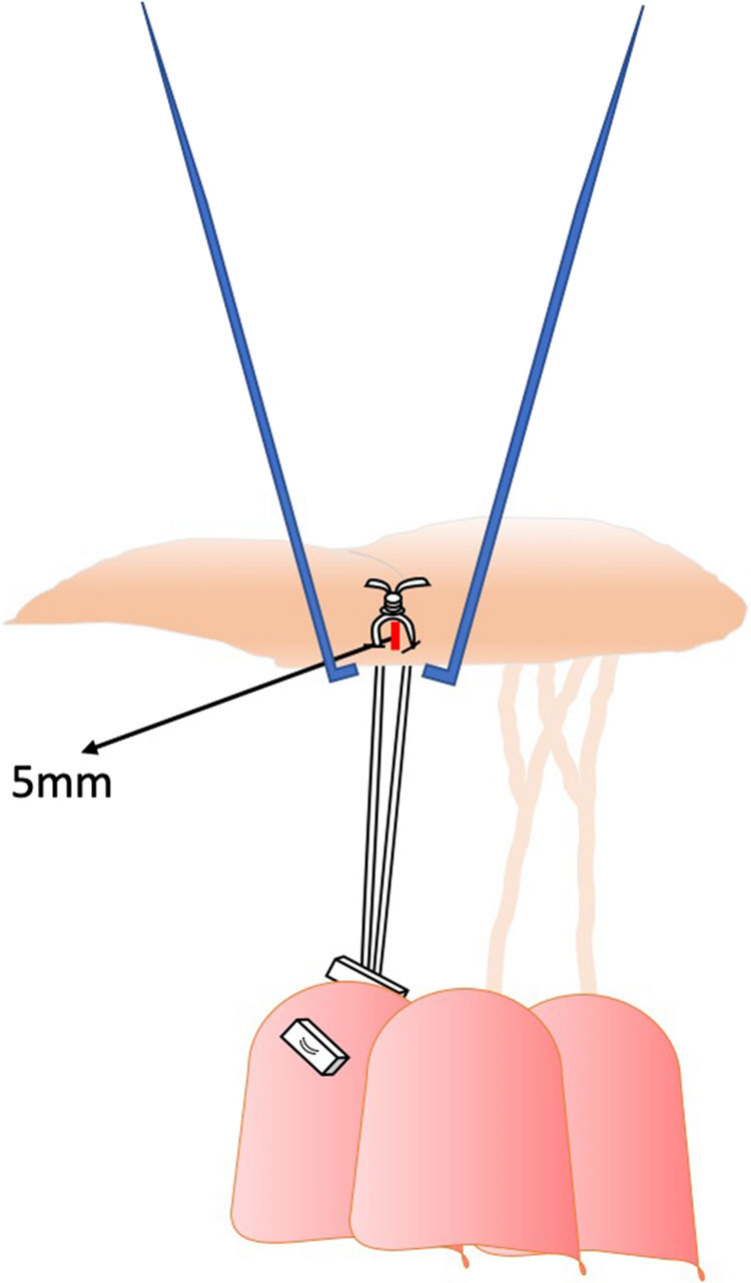
Calafiore^21^ proposed to pull the anterior leaflet (AL) with nerve hooks up to its maximum length and then to tie the artificial chord adding 5 mm to the border of the AL.

A) **Fixed chordal length with caliper**: von Oppell and Mohr measured the loop length^22^ taking into account the distance between the adjacent normal valve segment and the respective PM tip. Once the length is established, the surgeon constructs the loop using this fixed distance by means of a caliper. Then, the loop is sutured to the ventricular face of the leaflet‐free edge by a Gore‐Text suture passed inside the loop itself, while the two arms of the suture are passed inside the PM head and tied on two pledgets (Figure 1).
  Different use of the caliper was proposed by Doi et al.^27^ They passed the Gore‐tex suture through the rough area of the leaflet, from the atrial to the ventricular face, and then through the leaflet free edge. This leaves an adjustable loop, into which the surgeon introduces the caliper set at a distance already fixed by preoperative transesophageal echocardiography (Figure 2).
  Matsui et al.^29^ introduced a new device consisting of two small metal tubes with distal tips, one sliding over the other, to be used as a caliper. The exact length is determined by measuring the distance between the leaflet edge and the implantation site of the artificial cords on the papillary muscle based on an adjacent segment of normal valve. The Gore‐Tex suture can be tied without the knot slipping.
  Tam et al.^30^ proposed to roll a 4‐0 ePTFE suture around a caliper at a fixed length and then securing the loops with a 5‐0 ePTFE suture and on the PM tips using two pledgets.
B) **Fixed chordal length without caliper**: A series of inverted tight knots corresponding to a certain length^31^ are used instead of caliper to determine the appropriate chordal length. Other surgeons tie the loops to a predetermined length by temporarily securing them to a slit tube^32^ (Figure 3), a tourniquet,^33^ or plastic a tube.^34^ Chan et al.^35^ proposed to mark the fixed length (based on the length of a normal chord) on the ePTFE artificial chord and then to use an emoclip to hold the length while suturing (Figure 4).
C) **Anatomic chordal length**: All of the techniques mentioned involve measuring the length of new chords based on anatomically healthy chords, but it is important to keep in mind that surgeons work on the mitral valve when the heart is arrested in diastole, so this length may not mirror the chordal length on beating heart. Indeed, to overcome this possible distortion, Calafiore^21^ proposed pulling the anterior leaflet (AL) with nerve hooks to its maximum length and then tying the artificial cord by adding 5 mm to the edge of the AL (Figure 5).
  Alternatives approaches implicate tying cords after filling the left ventricle with saline, using a temporary Alfieri stitch^36^, ^37^ or emoclip^38^ to keep the leaflets coapting.
D) **Adjustable length**: Another key point is the knot slippage. Indeed, ePTFE sutures are very slippery, so the final length of the new cords may change while knotting. To avoid this possible inconvenience, some methods have been proposed.^20^, ^39^

**Figure 6:**
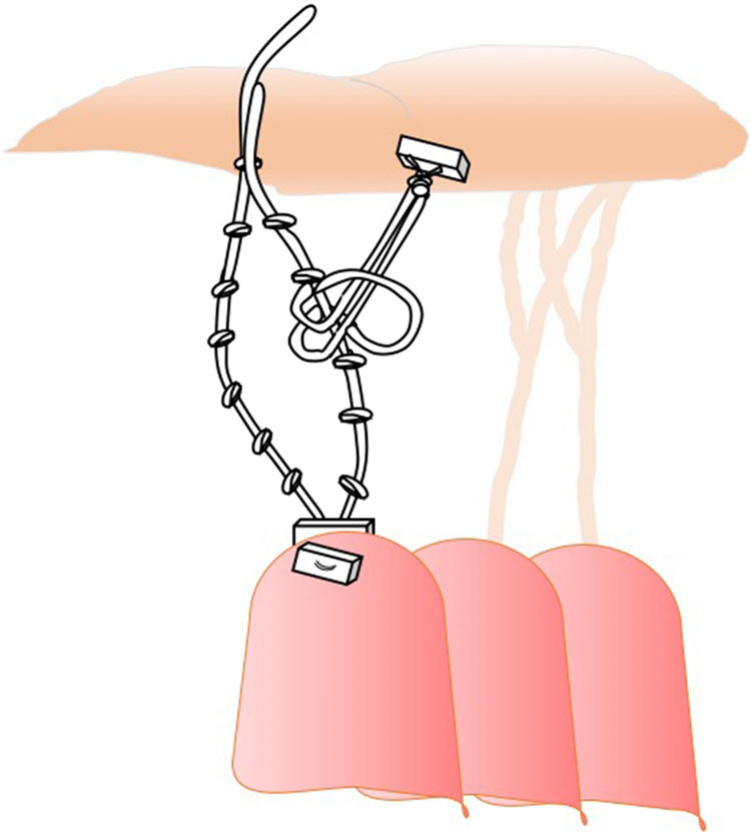
Maselli et al.^39^ proposed a tuneable loop technique (see text).

Maselli et al.^39^ proposed an adjustable loop technique composed of two parts: a papillary component with stop knots at constant intervals and a leaflet component with a reversible noose‐lace. After coupling of the two components to a presumably optimal length, a prosthetic loop is sutured in place. A hydrostatic test is then performed. The optimal cord length can be achieved by releasing the noose‐lace and sliding it over another fixing‐knot. Adjustment can be performed as often as necessary without stressing the anatomical structures (Figure 6).

**Figure 7:**
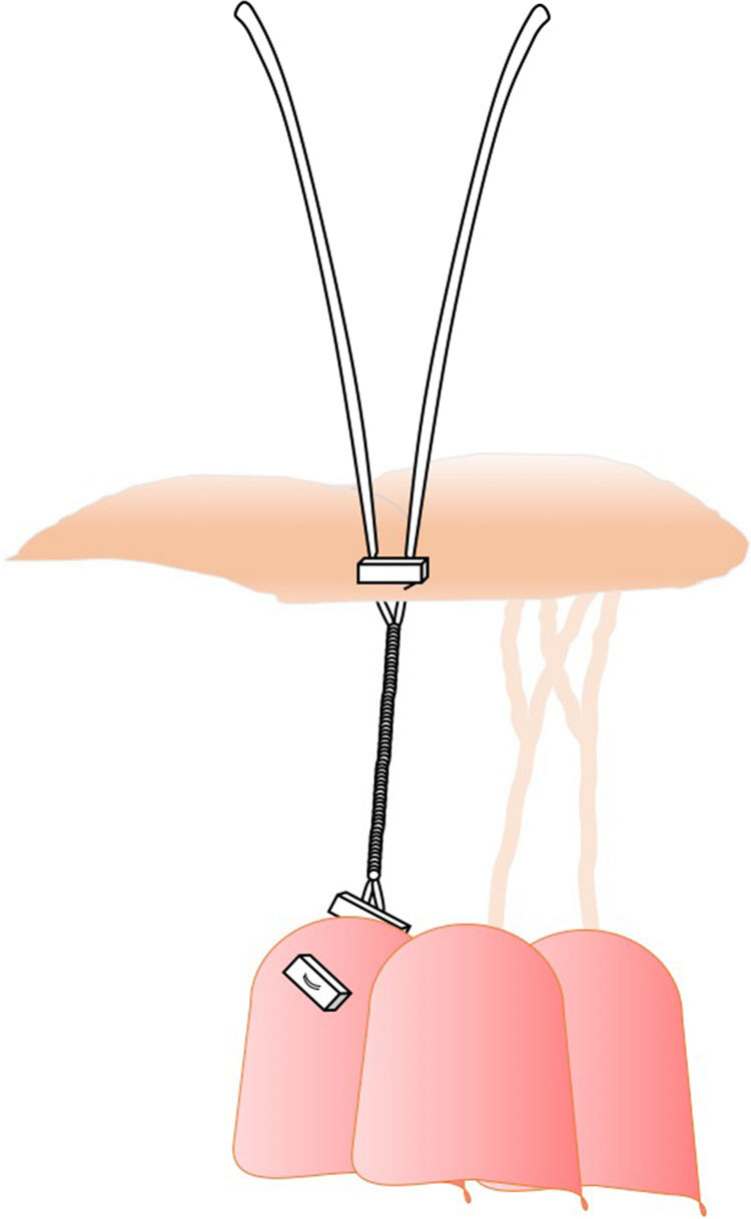
Shudo et al.^20^ proposed to tie multiple knots to a normal leaflet scallop so to calculate the number of knots to use in the prolapsing scallop, tying the suture only after filling test, to prevent knot‐slipping.

Another approach to prevent knot‐slipping is to tie multiple knots to a normal leaflet scallop to calculate the number of knots to be used in prolapsed scallop, tying the suture only after the filling test^20^ (Figure 7).

## 3 DRAWBACKS OF ARTIFICIAL CHORDS

MV repair is expected to last up to 20 years.^12^, ^13^, ^40^, ^41^ However, there are some drawbacks that surgeons need to be aware of when planning to perform MV repair.^42^ There are mainly three reasons why it might fail: technical failure, disease progression, or new disease (e.g., endocarditis).^43^ The symptomatology of patients facing failure of artificial cords varies. There are some reports in the literature regarding the presence of hemolytic anemia as a presentation of mitral valve repair failure, which could be due to the failure of artificial cords to endothelialize.^44^

First of all, the artificial chords could break over time,^45^, ^46^, ^47^ possibly due to hyalinization of the ePTFE pores or calcification. Therefore, surgeons should avoid pinching the ePTFE with forceps and clamps at the time of surgery.

The risk of MV repair failure is higher in the case of chordal rupture and is particularly high when thinner sutures (CV5)^48^ are used.

Another hot topic regarding the failure of artificial cords is the effect of left ventricular remodeling. It is possible that positive remodeling of the left ventricle after MV repair may cause a mismatch between artificial and native chords.^49^ Therefore, when faced with patients with extremely dilated left ventricles, surgeons should measure the artificial chords keeping in mind that left ventricular volumes may shrink over time.^50^

## 4 IS CHORDAL REPLACEMENT ONE‐SIZE‐FITS‐IN‐ALL APPROACH?

One of the main advantages of artificial chord implantation is their versatility. Hence, chordal replacement can be applied to almost any stage of degenerative MR including fibroelastic deficiency, single and bileaflet prolapse, and advanced Barlow's disease. In addition, they can be used extensive repair for hypertrophic cardiomyopathy or endocarditis.^51^

There is clear evidence that MV repair without tissue resection and extensive use of chordal replacement can be performed safely with excellent long‐term clinical results.

In a large meta‐analysis including 17 comparative studies of MVr with the “respect approach” (chordal replacement) versus the “resect approach” (leaflet resection), the former is associated with lower risk for operative mortality and pacemaker implantation, as well as larger annuloplasty rings and lower mean MV gradients. However, a word of caution is necessary; chordal replacement” cannot be a one‐size‐fits‐in‐all approach. Indeed, Dreyfus et al.^52^ advocated a complementary approach for posterior leaflet prolapse based on the concept “*respect when you can, resect when you should*”. These authors uses to address P2 height issue (with respect to P1 and P3) and excess PL width adding resecting approaches to artificial chordal replacement or native chordae transferring.^52^ This approach allow to avoid systolic anterior motion, especially in Barlow disease.

Other surgeons suggested to keep resecting, respecting, and respectful resection all in the surgeon's armamentarium so to use them according the MV disease to face with.^53^ In case of diffuse bileaflet disease or *forme fruste* degeneration of the posterior leaflet combined with elevated echocardiographic predictors of SAM (ie, acute aortomitral angle or narrow coaptation to septal distance), resection techniques are to be preferred. For focal disease of either leaflet, neochordal techniques can be the rightest choice. In case of diffuse posterior leaflet prolapse in combination with significant SAM predictors, a respectful resection technique has to be applied.

Conversely, in our philosophy, repair of the anterior leaflet using artificial chordae can be used in any type of prolapse, included, of course, Barlow's disease. The risk of SAM, which can happen when both leaflets meet inside the MV area, is generic, and is linked to the length of both leaflets (the longer the leaflets, the higher the risk) and to the surgical technique. Anytime the PL is mobile, the meeting point of the leaflets is inside the MV area and SAM can occur, especially when the PL or AL are longer than normal. If the PL is positioned in vertical position and motionless, as it happens when moderate overreduction is used or when artificial chordae are applied to PL for this purpose, SAM cannot occur, as the leaflets meet at the border of the MV area. Only a careful evaluation of length and mobility of both leaflet can prevent this dangerous complication.^54^

## 5 CONCLUSIONS

In the current era, MR should be treated with MV repair whenever possible. In addition to more classical corrective techniques, artificial chordae offer the surgeon a valuable tool to treat degenerative MR. Therefore, surgeons interested in MV repair should be familiar with artificial chordae to be able to tailor each repair to the individual mitral valve disease.
